# Factors Affecting Resilience of Nursing, Optometry, Radiography and Medical Laboratory Science Students

**DOI:** 10.3390/ijerph18083867

**Published:** 2021-04-07

**Authors:** Shirley Siu Yin Ching, Kin Cheung

**Affiliations:** School of Nursing, The Hong Kong Polytechnic University, Yuk Choi Road, Hung Hom, Kowloon, Hong Kong 100077, China; kin.cheung@polyu.edu.hk

**Keywords:** resilience, nursing, optometry, radiography, medical laboratory science, students, self-efficacy, mindfulness, coping

## Abstract

Background: The concern over the high level of stress experienced by students of the caring professions has led to increased attention being paid to the promotion of their resilience. Most earlier studies have focused on the resilience of medical and nursing students. There has been little exploration of the resilience and associated factors of students of other health-care disciplines. The aim of this study was to gather data from students of pre-registration health-care disciplines to identify the factors that influence their resilience. Method: Valid questionnaires were used to assess respondents’ resilience, self-efficacy, mindfulness, coping and trait positive and negative affect. The data were analyzed using descriptive statistics and univariate and general linear regression. Results: A total of 1320 university students from nursing, medical laboratory science, radiography and optometry were recruited. The results showed that the subjects’ resilience scores were lower than those of students in Western countries. We found self-efficacy and denial to be the common predictors for students of all disciplines. Conclusion: The resilience of students in the four disciplines was predicted by a combination of predictors. Faculties of universities and clinical mentors should collaborate in building resilience in their health-care students and support them to grow both personally and professionally during their careers.

## 1. Introduction

Health-care professionals play a key role in safeguarding the health of people in the community. However, the demands of time pressure, workload and emotional issues [1], as well as interacting with different professional groups and clients, frequently result in stress that impacts on their physical and mental wellbeing [2]. Some studies have found that health-care students reported high levels of perceived stress, which negatively affected their quality of life [2]. The academic aspect of their courses was a commonly reported stressor; examples included the time demands of the course and the perceived difficulty of their studies [3]. Clinical placement is an integral part of the health-care curriculum, but students are placed under considerable stress. Sources of stress can include the fear of unfamiliar situations arising, mistakes made with patients or in the handling of technical equipment [4], striving for learning opportunities and discovering the social rules in clinical settings [5]. Students are expected to prioritize the client’s needs before their own needs, and their own well-being is often ignored. This leads to the under-reporting of mental health conditions of health-profession students. Furthermore, the levels of psychological distress have been found to vary among health-care disciplines. For instance, nursing students were found to be substantially more distressed than physiotherapy and occupational therapy students during their programs [6]. On the other hand, dentistry students reported significantly higher stress than medicine, nursing, pharmacy and applied medical sciences students [7]. Concern over their well-being has led to increased attention being paid to the promotion of resilience across the health professions [8].

### 1.1. Studies into the Resilience of Health-Care Students

Studies of resilience began 50 years ago with children, adolescents, families and trauma survivors. The work on resilience in relation to health and illness began in the 1970s [2]. Resilience is the ability to recover, re-bound, bounce-back, adjust or even thrive following misfortune, change or adversity [9]. It was considered as an enduring personality trait in early studies but is viewed as a complex, dynamic and multi-dimensional phenomenon in contemporary studies [2].

Resilience is increasingly considered to be a critical capability of graduates. In a scoping review of student-resilience studies in health-professional education, it was found that about 80% of these studies were conducted in Europe and North America and published after 2011 [8]. Medicine, nursing, psychology and general health sciences were the most commonly studied professions. There was much less attention paid to the resilience of physiotherapy, radiotherapy and social work students. No articles related to optometrists were found. Very little research has been conducted on more than one health discipline [1], and thus the comparison of findings across disciplines is very limited. One study reported no differences between the resilience scores of medical and nursing students [10]. Comparing disciplines, resilience scores decreased in the order of health sciences, medical, dentistry and pharmacy [11]. Differences in students’ resilience scores occurred across countries. Medical and nursing students in Finland scored the highest, followed by those in China and then Japan [10]. Resilience was found to be linked with academic burnout, psychological well-being [12], student health and attrition [8].

### 1.2. Factors Affecting the Resilience of Health-Care Students

The existing literature clearly indicates the importance of enhancing the resilience of students across multiple health-care professions [8]. To examine the key psychological constructs affecting the health-care workforce, Rees and colleagues proposed the International Collaboration of Workforce Resilience model based on the empirical findings of previous studies and psychological models [13]. In response to workplace stressors, resilience was found to be associated significantly with mindfulness, self-efficacy, coping and psychological adjustment. Mindfulness is the awareness that arises from paying attention to one’s purpose in the present moment, non-judgmentally [14]. It is characterized by an ability to attain a de-centered perspective on events and a tendency to respond with flexibility to negative thoughts and emotions [15]. A positive association between mindfulness and resilience was identified in nursing students [16,17]. A mindful and accepting orientation promotes psychological resilience. Self-efficacy is an individual’s belief that they can perform a selected task [18]. According to social cognitive theory, human behavior is a continuous interaction between cognitive, behavioral and environmental factors. Cognitive events are altered by experiences of mastery from success. Efficacy expectations determine how much effort individuals will expend in the face of adversities [18]. Self-efficacy has been found to be correlated with resilience in nursing students [19]. Coping is a process of adjustment following an adverse event. There are three basic styles of coping with stress: task-oriented (i.e., making an effort to solve a problem), emotion-oriented (i.e., reducing emotional tension connected with a stressful situation) and avoidance-oriented (i.e., tendency to refrain from facing a stressful situation) [20]. Positive associations have been found between the use of problem solving/active coping and resilience in college students [21], nurses [22] and doctors [23]. Positive and negative affects, similar to other traits, are stable characteristics [24]. Positive affects broaden individuals’ capabilities for thoughts and actions and enhance personal resources; thus, they can lead to better life outcomes [25]. Negative affects can narrow attention and cognition by focusing on a specific threat or by promoting avoidance behaviour [26]. Trait negative affect has been associated with various negative psychological outcomes [27]. Following the International Collaboration of Workforce Resilience model and findings from existing studies, it was hypothesized in this study that mindfulness, self-efficacy, active coping and positive and negative affects would be associated with the resilience of health-care students.

### 1.3. Hong Kong Context

Health-care bachelor/pre-registration programs are offered by several government-funded or self-financed tertiary institutions in Hong Kong. The curriculum (including theoretical input and clinical practice requirements) and registration for most health-care disciplines are governed by statutory boards/councils [28]. The durations of the programs are four or five years; this is longer than the average of three years in the United Kingdom and Australia and four years in the United States. In contrast to Western countries, students are admitted directly to discipline-specific programs in Hong Kong without going through a foundation program and choosing the discipline after studying key health-science subjects. The discrepancies in durations and curriculum structures may result in differences in the stressors and responses of the health-care students in Eastern and Western countries. Several studies have been conducted on the resilience and psychological well-being of nursing students in Hong Kong, but none have investigated the resilience of radiography, optometry or medical laboratory science students. No significant differences were found in the resilience levels of junior and senior undergraduate students [29]. Neuroticism and emotional-oriented coping were identified as predictors of stress and burnout and psychological morbidity [30] while resilience predicted a positive mental status [31] and perceived well-being [29]. Resilience plays a protective role when nursing students face the demands of study. However, the factors associated with the resilience of nursing and other health-care students, such as those of optometry, radiography and medical laboratory science, in Hong Kong have not been explored. Culture has a significant impact on coping and resilience. While resilience in health care has been researched in Western cultures, there is a paucity of data in Asia pertaining to this factor [32]. Therefore, this study was conducted with students of pre-registration nursing and health-care disciplines. The objectives were (1) to investigate their levels of resilience and (2) to compare the key factors (i.e., mindfulness, self-efficacy, coping and trait positive and negative affect) that influence resilience in students of nursing and health-care disciplines.

## 2. Methods

### 2.1. Study Design and Subjects

This was a cross-sectional survey study using a convenience sampling method to collect data from health-care students studying in a Hong Kong university. The data collection was completed in March 2017. Only students enrolled in pre-registration baccalaureate programs in their health-care disciplines were included; those studying pre-registration programs at a Masters level were excluded. Institutional ethical clearance was obtained (HSEAR20150312001). With the permission of the school, health-care students were invited to complete the study questionnaire in their classroom settings after having the purpose of the study explained to them. The submission of a completed questionnaire implied that the students consented to participate in the study.

### 2.2. Instruments

The data were collected through a self-report survey which consisted of reliable and validated scales. The dependent variable, resilience, was measured by the 10-item short version of the Connor–Davidson Resilience Scale (CD-RISC). This instrument aims to measure an individual’s stress-coping ability and reactions to treatment for anxiety, depression and stress over the previous month [33,34]. Previous studies with community [35] and nursing populations [22] have reported that this scale was effective and dependable for measuring a spectrum of resilience in a normal clinical population [34]. A five-point Likert scale was adopted, with scores ranging from 0 = not true at all to 4 = true nearly all of the time. Summation of scores was used for analysis, with a higher score reflecting greater resilience [34]. Good validity was established on the CD-RISC; the correlation of the total items ranged from r = 0.30 to r = 0.70 [34], and the internal consistency was 0.90 [13]. In our study, α = 0.896.

Independent variables were measured as follows in the study questionnaire:

1. Demographic data included factors such as age, gender, marital status, discipline of study, year of study, cumulative grade-point average, living situation (alone/with family), religious belief, reasons for studying health care, any responsibility for dependent family members, financial assistance, any scholarship from the government or university and having a paid job.
2. The 10-item General Self-Efficacy Scale (GSE) is used to measure an individual’s ability to cope effectively with different stressful situations [36]. This scale employs a four-point Likert scale, with scores ranging from 1 = not at all true to 4 = exactly true. The summation score (10–40) was used for analysis, with a higher score indicating a greater sense of self-efficacy [36]. The internal consistency was found to be high, from α = 0.76 to α = 0.90 [13,36]. The GSE has good validity and has been found to be correlated positively with self-esteem and optimism and negatively with depression, loneliness, anxiety, shyness and pessimism in both men and women [37]. In our study, α = 0.866.
3. The 12-item Cognitive and Affective Mindfulness Scale, Revised (CAMS-R) measures a vast conceptualization of mindfulness that represents factors of awareness, internal experience, present moment focus, attention control and acceptance of experience [38]. In this study, the participants were asked to rate their responses on a four-point Likert scale, with scores ranging from 1 = rarely/not at all to 4 = almost always. Items 2, 6 and 7 were reverse-scored. Score summation was used for analysis, with a higher score representing higher levels of mindfulness consciousness. The discriminant validity of CAMS-R is established with the concurrent use of mindfulness, distress, well-being, emotion-regulation and problem-solving approaches [38]. Its internal consistency was found to be acceptable: α = 0.76 [38] and 0.80 [13]. In our study, α = 0.660.
4. The 20-item Positive and Negative Affect Scale (PANAS) measures participants’ overall emotional states at a particular time [24], with 10 items each for positive affect (PA) and negative affect (NA). A five-point Likert Scale is used, with scores ranging from 1 = very slightly or not at all to 5 = extremely. In the present study, the time instruction “generally” was used to measure trait-based affectivity for trait positive affect (TPA) and trait negative affect (TNA). The summation of scores (10–50) was used for analysis; for TPA, higher scores represented higher levels of positive affect, while for TNA, higher scores indicated more negative moods. The PANAS has been used frequently in research and clinical settings [24]. It demonstrates an excellent internal consistency ranging from α = 0.86 to α = 0.90 for PA and from α = 0.84 to α = 0.87 for NA [13,24]. In our study, α = 0.897 for PA and α = 0.873 for NA.
5. The 28-item Brief Cope Scale (BCS) measures adaptive and maladaptive coping skills using a four-point Likert scale (1 = I have not been doing this at all, and 4 = I have been doing this a great deal) [39]. These items were categorized into 14 scales (with two items each). The internal consistency for the 14 scales was as follows: active coping (α = 0.76, 0.68), planning (α = 0.73, 0.73), positive reframing (α = 0.78, 0.64), acceptance (α = 0.70, 0.57), humor (α = 0.72, 0.73), religion (α = 0.83, 0.82), using emotional support (α = 0.73, 0.71), using instrumental support (α = 0.82, 0.64), self-distraction (α = 0.54, 0.71), denial (α = 0.62, 0.54), venting (α = 0.58, 0.50), substance use (α = 0.89, 0.90), behavioral disengagement (α = 0.71, 0.65) and self-blame (α = 0.72, 0.69) ([13] vs [39] for the alphas). A summation of each subscale was used for analysis in this study. Higher scores in that scale indicated a higher utilization of that specific way of coping. In our study, α ranged from 0.384 to 0.892.

### 2.3. Data Analysis

SPSS version 25 (SPSS Inc., Chicago, IL, USA) was used for the data analysis. Descriptive statistics such as means, standard deviations, frequencies and percentages were used to summarize the study variables. The dependent variable was tested for normality for different student groups. Skewness, kurtosis and QQ plots all indicated the data were normally distributed. Hot deck imputation was used to handle missing data (1.58%) in all scales. After descriptive statistical analyses, univariate linear regression was used to determine the correlation between dependent and independent variables. Then, the independent variables that had significant correlations with the dependent variable were further analyzed using general linear regression. A *p*-value greater than 0.05 was considered significant.

## 3. Results

### 3.1. Demographics of the Students

Of the 1927 eligible subjects, a total of 1320 participated in the study. They were from the disciplines of nursing (N = 1070, 81.1%), medical laboratory science (N = 133, 10.1%), radiography (N = 65, 4.9%) and optometry (N = 52, 3.9%). The average response rate was 68.5% (ranging from 29.5% to 100%). Table 1 shows the characteristics of the health-care students. The majority of them were single, aged from 19 to 22, were living with their families and did not have particular religious beliefs. In general, nursing and medical laboratory students were distributed more evenly than the others with respect to year of study. Comparing disciplines, post-hoc tests results showed the differences among the groups. More of the nursing students were female and older. More radiography students indicated a single reason for studying, and professional status was the choice of the majority. Compared with nursing students, more medical laboratory science students had family responsibilities for dependent family members but received less financial assistance (Supplementary Table S1).

### 3.2. Differences among Health Care Students in Five Study Scales

Table 2 shows that there were significant differences among health-care students in the majority of the study scales. The students’ resilience scores, in descending order, were medical laboratory science (mean = 24.19), radiography (mean = 23.75), nursing (mean = 23.29) and optometry (mean = 22.10), but these differences did not reach statistical significance. The mean self-efficacy score for all subjects was 26.98. Post-hoc test results showed that radiography students had higher self-efficacy scores than nursing and optometry students. Optometry students had more negative affect and less mindfulness than nursing and medical laboratory science students. In terms of coping strategies, nursing students used less denial than medical laboratory and radiography students, used more instrumental support than medical laboratory and optometry students and used more acceptance and religion than optometry students (Supplementary Table S2).

### 3.3. Predictors of Resilience among Health-Care Students

Univariate linear regressions were used to identify the factors correlated with resilience for the overall participants and different groups of students. In general, all four scales were significantly correlated with resilience for all groups, in addition to age (for overall participants), gender and financial assistance (for nursing students), year of study (for medical laboratory science students) and having a paid job (for optometry students) (Supplementary Table S3).

Table 3 shows the predictors of resilience for the overall participants and different groups of students using general linear regression. The predictors varied for different student groups. The effects of predictors and the variance (R^2^) among students of the four disciplines were as follows:

1. Total sample: nine predictors (R^2^ = 0.648): high self-efficacy (t = 13.44), positive mindfulness (t = 12.89), high positive affect (t = 6.17), low negative affect (t = −6.51), positive denial (t = 16.08), negative behavioral disengagement (t = −6.37), positive reframing (t = 2.50), positive humor (t = 4.12) and high acceptance (t = 2.70).
2. Nursing students: nine predictors (R^2^ = 0.658): high self-efficacy (t = 11.59), positive mindfulness (t = 12.81), high positive affect (t = 5.13), low negative affect (t = −5.19), positive denial (t = 14.81), negative behavioral disengagement (t = −6.17), positive reframing (t = 3.24), positive humor (t = 2.96) and positive acceptance (t = 3.28).
3. Medical laboratory students: six predictors (R^2^ = 0.629): high self-efficacy (t = 5.13), high positive affect (t = 2.86), low negative affect (t = −4.05), positive denial (t = 2.99), positive humor (t = 2.40) and low self-blame (t = −2.24).
4. Radiography students: three predictors (R^2^ = 0.532): high self-efficacy (t = 2.05), positive mindfulness (t = 2.84) and positive denial (t = 2.69).
5. Optometry students: four predictors (R^2^ = 0.755): high self-efficacy (t = 4.97), positive mindfulness (t = 2.03), active coping (t = 2.23) and positive denial (t = 2.44).

In summary, based on the independent variables, self-efficacy and denial were the two common predictors for all disciplines. Mindfulness was a predictor for the nursing, radiography and optometry students. Positive affect, negative affect and humor were common predictors for nursing and medical laboratory science students. Behavioral disengagement, positive reframing and acceptance were exclusive predictors for the nursing students. Active coping and self-blame were exclusive predictors for the optometry and medical laboratory students, respectively.

## 4. Discussion

This is the first study to compare the level of resilience and its predictors in pre-registration nursing, optometry, radiography and medical laboratory science students. The medical laboratory science students had the highest resilience score, followed by radiography, nursing and optometry students but the differences did not reach statistical significance. All the variables (i.e., general self-efficacy, mindfulness, coping and positive and negative affect) were significantly correlated with resilience. The predictors varied for different student groups. The resilience of students in the four disciplines was predicted by a combination of predictors. Self-efficacy and denial were the two common factors for all disciplines. In the following, we discuss the differences in the independent variables among the groups of students, the differences in levels of resilience and the predictors of resilience that were common for the four groups of students and those that were unique to specific groups.

### 4.1. Differences in Independent Variables in Health-Care Students

Comparing the groups, the radiography students scored higher for self-efficacy than the nursing and optometry students. “Professional status”, which the radiography students most commonly stated as their single reason for studying—unlike the nursing and optometry students, whose reasons were helping others, personal interest, etc.—may give a hint to explain this higher self-efficacy, because self-concept and professional knowledge are sources of self-efficacy for health-care providers [40]. More of the nursing students in this study were female. They used more emotional-focused coping (i.e., more acceptance and religion) than the optometry students. The nursing students also used more instrumental support, which may be emotional in terms of meaning [41], than the medical laboratory and optometry students. This is consistent with previous studies, in which female university students used more emotional-focused coping (i.e., seeking support and meaning-focused coping) because they were influenced more strongly by social and emotional contexts [42]. The optometry students experienced more negative affect and less mindfulness than the nursing and medical laboratory science students. This may have contributed to this group having the lowest resilience score of the four groups, even though there was no significant difference. The medical laboratory science students in this study had more dependent family responsibilities, but they received less financial assistance and exhibited more denial than the nursing students. Students with dependents required more time and effort to attend classes and became more distressed [43]. Self-blame was a predictor of medical laboratory science students’ resilience levels in this study. The relationships among roles or financial demands, the use of a self-blame strategy and resilience requires further exploration.

### 4.2. Differences in Resilience in Health-Care Students

Resilience is an essential capability for health-care professionals to survive in the workplace [44]. The differences in resilience scores for the students of the four disciplines did not reach statistical significance. The medical laboratory science students ranked highest in their resilience scores, followed by radiography, nursing and optometry in decreasing order. Compared with medical laboratory science and radiography courses (i.e., three years), the longer durations of undergraduate nursing and optometry programs (i.e., four years) may lead to higher stress. The nature of clinical work, with greater demands to optimize patient care in busy clinical settings [5] and develop relationships with clients during clinical practice [45], may increase stress for radiography, nursing and optometry students and impose greater demands on their resilience.

The resilience scores of the students in this study ranged from 22.10 to 24.19. There are no recommended cut-off scores for CD-RISC 10. When compared to the scores of health-care students in the existing studies, our subjects scored lower than medical students in Canada (mean = 28.8 for female, mean = 31.2 for male) [46] and nursing students in India (mean = 26.3) [17]. The CD-RISC 10 has been used in two studies in Hong Kong. The resilience levels of Chinese undergraduate and postgraduate students (who were staff nurses) were found to be 23.8 and 24.9, respectively [29], and male and female Chinese adolescent students had scores of 25.1 and 24.3, respectively [47]. These were similar to the scores of our subjects. In general, health-care students in Western countries have been found to have higher resilience levels than their counterparts in Hong Kong. There have been very few cross-cultural comparison studies of resilience. These differences may be explained by the busy working environments in the health-care settings in Hong Kong and the values and beliefs of Chinese people. In a study of trauma survivors, a lower level of dialectical thinking to reconcile opposing perspectives and higher independent self-construal in American trauma survivors affected the effectiveness of their responses in the aftermath of trauma and their construction of a more resilient self-concept than survivors in Hong Kong and China [48].

### 4.3. Similarities and Differences in Predictors of Resilience among Health-Care Students

Self-efficacy was one of the common predictors for all disciplines. This finding is consistent with that of a scoping review that identified self-efficacy to be one of the personal factors informing resilience enhancement in health professional students [8]. Individuals with high levels of perceived self-efficacy tend to conceptualize problems as challenges rather than as threats or uncontrollable situations, trust their own abilities and show perseverance in the face of adversity, experience less negative emotional arousal in demanding tasks and think in self-enhancing ways [18]. Their self-worth is not threatened by setbacks [49]. Self-efficacy is of specific significance in nursing and health-care students. In clinical placements, students with low self-efficacy will not initiate tasks that they are not sure about if they are concerned about the consequences in order to avoid making mistakes [50]. In this study, the mean self-efficacy score for the students was 26.98, which is similar to that of male (mean = 27.3) and female (mean = 25.1) nursing students in China [51] but lower than for a sample of nursing students (mean = 30.4) in the United Kingdom [52]. One review found that collective efficacy (i.e., a shared belief in a group’s combined capabilities to execute the courses of action required to produce a given attainment) in non-Western groups appears to operate in much the same way as self-efficacy operates for Western groups [53]. Chinese people’s beliefs in collaborative control have a significant influence on their behaviours. This may explain the lower self-efficacy scores of Chinese students.

An unexpected finding in this study was the use of denial as a common predictor, associated positively with resilience for all disciplines. Denial was categorized as an avoidance strategy [54], ineffective defense mechanism [55] or maladaptive strategy and was positively correlated with perceived stress [1] and self-blame in nurses [56]. A closer look at the two items of denial in the Brief Cope Scales—i.e., “I have been saying to myself that ‘this is not real’”, and “I have been refusing to believe that it has happened”—reflected detachment or keeping the problem at a distance. In studies of individuals with cancer or myocardial infarction, denial contributed to less negative affect [57] and reduced distress by protecting against overwhelming events and feelings due to its distractive effect [55]. Some studies with paramedic [58] and medical and nursing students [59] have indicated that they adopted detachment strategies to manage their emotions. Emotional detachment may be desirable in professionals involved in distressing scenes, and denial of negative feelings is a short-term measure for extreme situations [58], but long-term use can actually indicate maladaptive coping [60]. Thus, the effect of denial depends on whether it is unconscious or conscious, or a trait or state [55]; these were not explored in this study. However, the students’ use of denial may indicate that they were under considerable stress, which taxed their resilience and coping mechanisms.

Mindfulness was a predictor of resilience for the students in most of the disciplines (i.e., nursing, radiography and optometry). This is consistent with previous research findings with nursing students [17]. Mindfulness is the awareness that arises as a result of paying attention to one’s purpose in the present moment, non-judgmentally [14]. Mindfulness facilitates emotional regulation and cognitive adjustment in times of stressful encounters. It can lead to a reduction in stress and self-doubt and an increase in self-compassion, self-awareness and empathy and can help to reduce burnout and emotional exhaustion as a result of caring activities [61]. As indicated in this study, mindfulness emerged as a predictor for nursing, radiography and optometry students whose courses included clinical components with direct patient care. A qualitative study of Hong Kong nursing students who scored highly in terms of resilience found that they had higher self-awareness and coped by separating themselves from situations in clinical placement; these are signs of mindfulness [5].

Trait positive and negative affect and humor emerged as common predictors for the nursing and medical laboratory science students. This echoes the finding reported by Loh, Schutte and Thorsteinsson that resilience was associated with the effects of positive affect on depression in psychology students [62]. A positive affect leading to the pursuit of effective coping resources, such as creative thoughts and actions at stressful times, can help individuals to transform negative emotional experiences to positive ones [63]. The infusion of positive meaning or problem-focused coping may have contributed to the students’ resilience. The inverse relationship of trait negative affect and resilience is consistent with existing studies with student nurses [13] and health professionals [64]. Individuals develop negative emotions to protect themselves in times of stress, but a narrowing of cognition and attention may lead to fewer resources and poorer life outcomes [62]. Humor was positively correlated to resilience in this study. Similarly, an earlier study found that emergency service personnel used this technique successfully to cope with stressful situations. In particular, it helped them to distance themselves from stress sources, indicating a cognitive shift to less threatening perspectives on the situation [65]. Humor has also been shown to regulate negative emotions and enhance positive ones, thus creating psychological flexibility and building resilience to future stressful life events [65].

Positive reframing, acceptance and behavioural disengagement were exclusive predictors for nursing students in this study. Compared with the other disciplines, the nature of nursing work is characterized by more direct contact with patients with various health conditions, ranging from critical to acute and to chronic, in busy clinical settings. Nursing requires shift work and interactions with members of multidisciplinary health-care teams. Positive reframing and acceptance are examples of adaptive strategies [60]. Reframing burdensome experiences was found, in a review study, to contribute to the development of resilience in nursing students [66]. Meaning-making, as a form of positive reframing, was used by nursing students in Hong Kong to enable them to cope sustainably and reduce cognitive or psychological disturbances during clinical placements [5]. Active acceptance, in contrast to resigned acceptance, refers to facing reality even if it does not fit one’s expectations or desires and the willingness to deal with this reality and not allow it disrupt one’s life. It is a form of emotionally focused coping, which is especially relevant in the face of unchangeable and uncontrollable situations [67] such as demands in busy clinical settings. Behavioral disengagement is a less adaptive strategy [60]. It was negatively associated with the nursing students’ resilience in this study, which may have indicated their avoidance of stressful encounters; this phenomenon requires more attention from academic and clinical staff than it is given currently.

Active coping was the exclusive predictor for optometry students. Active coping has been categorized previously as problem-focused coping [60]. A review found that problem solving and taking action were consistently reported as signs of resilience in health-profession students [8] and components of resilience building intervention for nurses [68]. Problem-focused coping contributes to resilience by eliminating the stressor and promoting positive thinking and thinking avoidance when a problem is unsolvable [69].

Self-blame was the exclusive predictor associated negatively with the resilience of the medical laboratory science students. It has been categorized as an emotion-focused coping [69] or avoidance coping [70] mechanism. An earlier quantitative study with Japanese nursing students found a negative association between self-blame and their general health [70]. In a qualitative study, self-blame reinforced nursing students’ negative anticipation, undermined their confidence when they conformed to external requirements during clinical placement and contributed to low resilience and high burnout [5]. On the contrary, Gibbon found that those that scored highly in self-blame were likely to set high goals and take responsibility for working diligently, which resulted in achievement [71]. The vast majority (78.7%) of the medical laboratory science students in this study scored high grade point averages (i.e., ≥3 out of 4). The association of high achievement goals and the use of a self-blame strategy was not explored in this study.

Age and gender were not identified as predictors of resilience in this study. Similarly, no consistent conclusions have been drawn from existing findings. Some found that older students [29,72] and male students had higher resilience scores [11,73], but others found no differences in age [73] or gender [72].

### 4.4. Implications

The need for a focus on resilience across the health professions has been advocated over the past decade [2]. However, most studies of resilience have recruited medicine, nursing, and psychology students [8]. The findings of this study gave hints about resilience and its predictors for optometry, radiography and medical laboratory science students as well as nursing students. Supporting their self-efficacy, mindfulness, coping (i.e., positive reframing, acceptance and humor), promoting positive affect and reducing negative affect may increase the resilience of these students, as indicated by the findings of this study.

It is important for university teachers to understand the sources of stress (e.g., academic, clinical, personal and financial) that may tax the resilience of their health-care students in greater depth. Teachers and clinical mentors are encouraged to support these students to reflect on their expectations about their studies and clinical placements, help them to reframe their experiences by identifying meaning in them, facilitate flexible use of internal and external coping resources such as social support and allow time for self-reflection and self-care to enhance their resilience [5].

The implementation of resilience programs in undergraduate curricula [2] can facilitate the holistic development of students and help them to cope better with their busy schedules. To increase self-efficacy, students must experience success with the tasks they might have expected to fail by practicing clinical skills under supervision, receiving continuous feedback throughout clinical placements and acting independently when they reach a certain level of competency [50]. Engagement in innovative learning strategies, such as interprofessional learning [74] and problem-based learning [75], can increase students’ decision-making and problem-solving skills and subsequently increase their self-efficacy. Mindfulness-based training, permeated with elements of flexibility, self-discovery, self-compassion, and empowerment, with the aim of generating a natural transfer of the skills developed in meditation to their studies, decision making and relationships, is one way to optimize the wellbeing and resilience of students [76]. In addition, Grant and Kinmann recommended the development of emotional-resilience curricula in students of the caring professions, including reflective practice on emotional reactions and beliefs, supervision that facilitates the discussion of emotional reactions to practice, peer coaching for stress management and experiential learning to enhance the effective use of emotions to facilitate problem solving [77].

The findings of this study draw our attention to the use of avoidance-coping strategies by nursing students. Behavioral disengagement was a negative predictor of resilience. Although the use of acceptance and denial in the short term were positively correlated with resilience, our results suggest that nursing students may be coping with negative emotions in the face of unchangeable and uncontrollable situations, which are probably associated with clinical practice. They could benefit from being given special support before their clinical placements, such as briefings, orientation or sharing of experience by senior year students and reserving “protected time” for clinical teachers to coach and debrief them and allow them to reflect on their performance.

Further investigation is recommended into health-care students’ reasons for using denial and self-blame. Further study of their resilience mechanisms is also recommended. Individual factors were included as independent variables in this study. Further studies may be conducted to investigate the effects on health-care students’ resilience regarding some contextual factors, such as support from the workplace, work culture and other individual factors such as professional identity [1], self-concept [40] and body image [78]. Furthermore, since most previous studies have focused on resilience at the individual level [79], a future research direction would be to explore and support health-care students and professionals as a group in response to emerging health-care demands and the expansion of professional roles.

### 4.5. Limitations

There were 1320 health-care students who participated in this study. The sample included medical laboratory science, radiography and optometry students whose resilience and coping have received little attention in previous research. However, the data collection was conducted in only one university, so the generalizability to other institutions may be questionable. Only third-year radiography and optometry students were recruited for this study. The specific study and clinical demands and the accumulated experience and learning in the professions may have influenced the findings. Thus, the level of resilience and the predictors of junior and final-year radiography and optometry students may have been different. Future studies could include larger numbers of health-care students from all years of study, from a range of institutions and from other disciplines such as physiotherapy, occupational therapy, speech therapy, counseling and medicine. The cross-sectional design of this study may have limited the identification of changes in independent variables and resilience across time. A longitudinal and qualitative design would enable researchers to capture the changes in factors from junior to senior years of study and how these factors affect resilience in the contexts of their academic and personal lives.

## 5. Conclusions

Understanding the factors which influence resilience is the first step to understanding and developing interventions specifically for building resilience in nursing and other health-care students. This study found that the students’ resilience was predicted by a combination of factors, with self-efficacy and denial emerging as the common predictors for all disciplines. University teachers and clinical mentors should collaborate to build resilience in students in the caring professions, thus equipping them to face challenges and promote their wellbeing.

## Figures and Tables

**Table 1:** Characteristics of the health-care students.

Characteristics of Students	Total (N = 1320) n (%)	Nursing (N = 1070) n (%) (A)	Medical Laboratory Science (N = 133) n (%) (B)	Radiography (N = 65) n (%) (C)	Optometry (N = 52) n (%) (D)	Comparison among Different Groups of Students χ^2^, df, Phi, *p*	Post-Hoc Test # (A = Nursing; B = Medical Laboratory Science; C = Radiography; D = Optometry)
Age						109.63, 9, 0.29, *p* < 0.001 **	
≤18	68 (5.2)	49 (4.6)	18 (13.5)	1 (1.5)	0 (0.0)		(A,B) (B,C)
19–20	457 (34.7)	314 (29.4)	68 (51.1)	39 (60.0)	36 (69.2)		(A,B) (A,C) (A,D)
21–22	515 (39.1)	447 (41.9)	37 (27.8)	21 (32.3)	10 (19.2)		(A,B) (A,D)
≥23	277 (21.0)	257 (24.1)	10 (7.5)	4 (6.2)	6 (11.5)		(A,B) (A,C)
Gender						82.80, 3, 0.26, *p* < 0.001 **	
Male	380 (29.9)	261 (25.2)	47 (37.9)	47 (73.4)	25 (51.0)		
Female	891 (70.1)	773 (74.8)	77 (62.1)	17 (26.6)	24 (49.0)		(A,B) (A,C) (A,D) (B,C)
Marital status						4.15, 3, 0.06, *p* = 0.25	
Single	1298 (98.6)	1052 (98.5)	133 (100.0)	62 (96.9)	51 (100.0)		
Non-single	18 (1.4)	16 (1.5)	0 (0.0)	2 (3.1)	0 (0.0)		
Year of study						269.39, 9, 0.45, *p* < 0.001 **	
Year 1	243 (18.4)	200 (18.7)	43 (32.3)	0 (0.0)	0 (0.0)		(A,B)
Year 2	208 (15.8)	173 (16.2)	35 (26.3)	0 (0.0)	0 (0.0)		(A,B)
Year 3	481 (36.4)	322 (30.1)	42 (31.6)	65 (100)	52 (100)		
Year 4	388 (29.4)	375 (35.0)	13 (9.8)	0 (0.0)	0 (0.0)		(A,B)
Cumulative grade Point average						170.98, 9, 0.36, *p* < 0.001 **	
2–2.5	95 (7.3)	85 (8.1)	4 (3)	1 (1.6)	5 (9.6)		
2.6–3	550 (42.3)	496 (47.2)	24 (18.2)	14 (21.9)	16 (30.8)		(A,B)(A,C)
3.1–3.5	552 42.5)	429 (40.8)	65 (49.2)	40 (62.5)	18 (34.6)		(A,C)(C,D)
≥3.6	102 (7.9)	41 (3.9)	39 (29.5)	9 (14.1)	13 (25.0)		(A,B)(A,C)(A,D)
Living						5.24, 3, 0.06, *p* = 0.16	
With family	1213 (92.8)	983 (92.7)	127 (95.5)	61 (93.8)	42 (85.7)		
Alone	94 (7.2)	77 (7.3)	6 (4.5)	4 (6.2)	7 (14.3)		
Religious beliefs						4.92, 3, 0.06, *p* = 0.18	
No	956 (72.8)	761 (71.6)	101 (75.9)	53 (81.5)	41 (78.8)		
Yes	357 (27.2)	302 (28.4)	32 (7.2)	12 (18.5)	11 (21.2)		
Reasons of studying						25.30, 3, 0.14, *p* < 0.001 **	
One of the reasons a	339 (25.7)	270 (25.2)	26 (19.5)	33 (50.8)	10 (19.2)		
Multiple reasons b	981 (74.3)	800 (74.8)	107 (80.5)	32 (49.2)	42 (80.8)		(A,C)(B,C)(C,D)
Family responsibility						18.98, 3, 0.12, *p* < 0.001 **	
None	1080 (82.1)	899 (84.3)	96 (72.2)	48 (75.0)	37 (71.2)		
Dependent c	235 (17.9)	167 (15.7)	37 (27.8)	16 (25.0)	15 (28.8)		(A,B)
Financial assistance						13.93, 3, 0.10, *p* = 0.003 **	
No	727 (55.3)	563 (52.9)	88 (66.2)	41 (64.1)	35 (67.3)		
More than one source of assistance	587 (44.7)	502 (47.1)	45 (33.8)	23 (35.9)	17 (32.7)		(A,B)
Scholarship from government						10.57, 3, 0.09, *p* = 0.01 *	
No	1259 (96.4)	1027 (97.1)	127 (95.5)	57 (90.5)	48 (92.3)		
Yes	47 (3.6)	31 (2.9)	6 (4.5)	6 (9.5)	4 (7.7)		(A,C)
Scholarship from university/hospital						50.21, 3, 0.20, *p* < 0.001 **	
No	1195 (91.5)	994 (94.0)	103 (77.4)	56 (88.9)	42 (80.8)		
Yes	111 (8.5)	64 (6.0)	30 (22.6)	7 (11.1)	10 (19.2)		(A,B)(A,D)
Paid job						5.24, 3, 0.06, *p* = 0.16	
No	506 (38.5)	406 (38.1)	47 (35.3)	33 (51.6)	20 (38.5)		
Yes	808 (61.5)	659 (61.9)	86 (64.7)	31 (48.4)	32 (61.5)		

Note: ^a^ One of these reasons: helping others, family influence, professional status, stable income, personal interest. ^b^ Multiple reasons: more than one reason. ^c^ Dependent(s) to be taken care of included a husband, wife, partner, children, disabled parents, other relatives etc. * *p* < 0.05; ** *p* < 0.01. # Pairwise Z-tests with Bonferroni adjustment.

**Table 2:** Comparison among health care students in five study scales by one-way ANOVA.

Study Scales	Total (N = 1320) Mean, SD	Nursing (N = 1070) Mean, SD (A)	Medical Laboratory Science (N = 133) Mean, SD (B)	Radiography (N = 65) Mean, SD (C)	Optometry (N = 52) Mean, SD (D)	One-Way ANOVA	Post-Hoc Test # (A = Nursing; B = Medical Laboratory Science; C = Radiography; D = Optometry)
CD-RISC	23.35, 5.43	23.29, 5.32	24.19, 5.22	23.75, 6.45	22.10, 6.63	F(3, 1316) = 2.15, *p* = 0.09	
GSE	26.98, 3.75	26.85, 3.69	27.65, 3.86	28.43, 3.97	26.19, 3.94	F(3, 1316) = 5.89, *p* = 0.001 **	(A,C)(C,D)
CAMS-R	31.93, 3.48	31.98, 3.45	32.37, 3.63	31.43, 3.23	30.52, 3.72	F(3, 1316) = 4.10, *p* = 0.007 **	(A,D)(B,D)
PANAS							
PAS	27.25, 6.43	27.36, 6.37	26.94, 6.80	26.11, 7.18	27.08, 5.80	F(3, 1316) = 0.90, *p* = 0.44	
NAS	22.04, 7.60	21.94, 7.41	21.07, 8.08	22.69, 9.04	25.75, 7.31	F(3, 1316) = 5.13, *p* = 0.002 **	(A,D)(B,D)
BCS							
Self-distraction	5.53, 1.04	5.53, 1.01	5.65, 1.10	5.43, 1.05	5.33, 1.38	F(3, 1316) = 1.49, *p* = 0.22	
Active coping	5.61, 0.87	5,63, 0.85	5.59, 0.90	5.52, 0.90	5.38, 1.12	F(3, 1316) = 1.51, *p* = 0.21	
Denial	4.99, 1.07	4.94, 1.04	5.23, 1.16	5.37, 1.21	4.90, 1.12	F(3, 1316) = 5.76, *p* < 0.001 **	(A,B)(A,C)
Substance use	2.85, 1.40	2.82, 1.38	2.70, 1.33	3.78, 1.72	2.65, 1.06	F(3, 1316) = 10.90, *p* < 0.001 **	(A,C)(B,C)(C,D)
Use of emotional support	5.40, 1.19	5.44, 1.17	5.23, 1.19	5.40, 1.16	5.08, 1.57	F(3, 1316) = 2.48, *p* = 0.06	
Useof instrumental support	5.47, 1.23	5.53, 1.18	5.22, 1.39	5.31, 1.24	5.04, 1.53	F(3, 1316 )= 5.30, *p* = 0.001 **	(A,B)(A,D)
Behavioral disengagement	4.11, 1.28	4.08, 1.26	4.18, 1.35	4.52, 1.32	3.94, 1.14	F(3, 1316) = 2.87, *p* = 0.04 *	(A,C)
Venting	5.07, 1.10	5.09, 1.08	4.98, 1.24	5.17, 1.08	4.85, 1.09	F(3, 1316) = 1.24, *p* = 0.29	
Positive reframing	5.38, 1.02	5.40, 1.00	5.35, 1.17	5.37, 0.98	5.15, 1.09	F(3, 1316) = 1.01, *p* = 0.39	
Planning	5.32, 0.97	5.32, 0.95	5.28, 1.08	5.46, 0.94	5.17, 1.13	F(3, 1316) = 0.94, *p* = 0.42	
Humor	4.82, 1.31	4.83, 1.30	4.73, 1.44	5.02, 1.17	4.52, 1.38	F(3, 1316) = 1.62, *p* = 0.18	
Acceptance	5.71, 0.96	5.74, 0.91	5.64, 1.09	5.71, 1.01	5.33, 1.32	F(3, 1316) = 3.31, *p* = 0.02 *	(A,D)
Religion	4.41, 1.63	4.48, 1.63	4.14, 1.68	4.48, 1.45	3.71, 1.50	F(3, 1316) = 5.06, *p* = 0.002 **	(A,D)
Self-blame	4.79, 1.26	4.79, 1.23	4.65, 1.42	5.09, 1.17	4.77, 1.46	F(3, 1316) = 1.78, *p* = 0.15	

Note: Positive and Negative Affect Scale (PANAS); General Self-Efficacy Scale (GSE); Cognitive and Affective Mindfulness Scale, Revised (CAMS-R); Connor–Davidson Resilience Scale (CD-RISC); Brief Cope Scale (BCS); * *p* < 0.05; ** *p* < 0.01. # Tukey’s HSD (honest significant difference) test.

**Table 3:** General linear regressions to determine the predictors of resilience in all students and four student groups (N = 1320).

Variables	Total (N = 1320) R^2^ 0.648			Nursing (N = 1070) R^2^ 0.658			Medical Laboratory Science (N = 133) R^2^ 0.629			Radiography (N = 65) R^2^ 0.532			Optometry (N = 52) R^2^ 0.755		
	B	95% CI	*p*	B	95% CI	*p*	B	95% CI	*p*	B	95% CI	*p*	B	95% CI	*p*
Age (≥23 as ref.)															
≤18	0.50	−0.36, 1.37	0.25												
19–20	0.49	0.01, 0.98	0.05												
21–22	0.35	−0.13, 0.82	0.15												
≥23 as ref															
Gender Male				−0.07	−0.52, 0.39	0.77									
Female as ref.															
Year of study															
Year 1							−1.60	−3.69, 0.49	0.13						
Year 2							−2.11	−4.28, 0.05	0.06						
Year 3							−1.08	−3.19, 1.04	0.32						
Year 4 as ref.															
Financial assistance More than one source of assistance as ref. No				0.24	−0.14, 0.62	0.22									
Paid Job Yes as ref. No													−1.09	−3.38, 1.21	0.34
GSE	0.40	0.34, 0.45	<0.001 **	0.38	0.32, 0.45	<0.001 **	0.44	0.26, 0.61	<0.001 **	0.37	0.01, 0.73	0.05 ^a^*	0.84	0.50, 1.18	<0.001 **
CAMS-R	0.41	0.34, 0.47	<0.001 **	0.44	0.37, 0.51	<0.001 **	0.15	−0.07, 0.36	0.18	0.63	0.19, 1.07	0.006 **	0.37	0.00, 0.73	0.05 ^b^*
PANAS															
PAS	0.10	0.07, 0.13	<0.001 **	0.09	0.06, 0.13	<0.001 **	0.13	0.04, 0.23	0.005 **				0.21	−0.01, 0.44	0.06
NAS	−0.09	−0.12, −0.06	<0.001 **	−0.08	−0.11, −0.05	<0.001 **	−0.17	−0.25, −0.09	<0.001 **	−0.08	−0.21, 0.06	0.25	−0.13	−0.28, 0.02	0.09
BCS															
Self-distraction	0.10	−0.09, 0.29	0.31	0.06	−0.15, 0.27	0.57	0.38	−0.21, 0.97	0.20				0.27	−0.65, 1.20	0.55
Active coping	0.07	−0.17, 0.32	0.56	−0.21	−0.49, 0.07	0.14	0.48	−0.27, 1.22	0.21	1.04	−0.36, 2.43	0.14	1.25	0.12, 2.38	0.03 *
Denial	1.51	1.33, 1.70	<0.001 **	1.56	1.35, 1.77	<0.001 **	0.80	0.27, 1.33	0.003 **	1.46	0.37, 2.54	0.009 **	1.17	0.20, 2.14	0.02 *
Substance use															
Use of emotional support	−0.13	−0.35, 0.08	0.22	−0.06	−0.29, 0.18	0.64							−0.62	−1.74, 0.51	0.28
Use of instrumental support	0.01	−0.20, 0.22	0.92	−0.03	−0.26, 0.21	0.83							−0.27	−1.44, 0.90	0.64
Behavioral disengagement	−0.52	−0.68, −0.36	<0.001 **	−0.57	−0.75, −0.39	<0.001 **									
Venting															
Positive reframing	0.29	0.06, 0.52	0.01 *	0.42	0.16, 0.67	0.001 **	−0.12	−0.79, 0.56	0.74						
Planning	0.08	−0.15, 0.32	0.48	0.17	−0.10, 0.43	0.21				−0.39	−1.88, 1.11	0.60			
Humor	0.33	0.17, 0.49	<0.001 **	0.26	0.09, 0.45	0.003 **	0.58	0.10, 1.06	0.02 *	0.57	−0.62, 1.76	0.34	0.19	−0.70, 1.08	0.68
Acceptance	0.33	0.09, 0.57	0.007 **	0.45	0.18, 0.72	0.001 **				1.00	−0.42, 2.42	0.16	−0.25	−1.52, 1.02	0.70
Religion	−0.06	−0.18, 0.06	0.31	−0.11	−0.24, 0.02	0.10	0.11	−0.25, 0.48	0.54						
Self-blame	−0.05	−0.22, 0.12	0.54	0.06	−0.13, 0.25	0.56	−0.48	−0.91, −0.06	0.03 *				0.09	−0.71, 0.88	0.83

Note: Positive and Negative Affect Scale (PANAS); General Self-Efficacy Scale (GSE); Cognitive and Affective Mindfulness Scale, Revised (CAMS-R); Connor-Davidson Resilience Scale (CD-RISC); Brief Cope Scale (BCS). ^a^ *p* = 0.045; ^b^ *p* = 0.049; * *p* < 0.05; ** *p* < 0.01.

## Supplementary Materials

The following are available online at https://www.mdpi.com/article/10.3390/ijerph18083867/s1, Table S1: Summary of Chi-square and Pairwise Z-Tests. (Only significant pairs were shown), Table S2: Summary of One-way ANOVA and Post hoc Tukey test. (Only significant pairs were shown), Table S3: Univariate linear regressions to determine the correlation between independent variables and resilience in all students and four student groups (N = 1320).
