# Supplementary material for: Factors Affecting Resilience of Nursing, Optometry, Radiography and Medical Laboratory Science Students
Source: Int J Environ Res Public Health. 2021 Apr 7;18(8):3867. doi: 10.3390/ijerph18083867 (PMC8067740; doi:10.3390/ijerph18083867)
Supplement: Supplementary file 1 [file ijerph-18-03867-s001.pdf]

Supplementary Table S1. Summary of Chi-square and Pairwise Z-Tests. (Only significant pairs were shown)

Chi-square and Post hoc test*<br><br>Characteristics of Students	University Nursing (N=1070)<br><br>(A)	University Medical Laboratory Science (N=133)<br>(B)	University Radiography (N=65)<br><br>(C)	University Optometry (N=52)<br><br>(D)	Comparison among different groups of students<br><br>χ^2, df, Phi, p
Age					
≤ 18	B(.000)	A(.000)<br>C(.021)	B(.021)		109.63, 9, 0.29, **p<0.001**
19-20	B(.000)<br>C(.000)<br>D(.000)	A(.000)	A(.000)	A(.000)	
21-22	B(.011)<br>D(.007)	A(.011)		A(.007)	
≥ 23	B(.000)<br>C(.005)	A(.000)	A(.005)		
Gender	B(.015)<br>C(.000)<br>D(.000)	A(0.015)<br>C(0.000)	A(.000)<br>B(.000)	A(.000)	82.80, 3, 0.26, **p<0.001**
Year of Study					
Year 1	B(.000)	A(.000)			269.39, 9, 0.45, **p<0.001**
Year 2	B(.004)	A(.004)			
Year 3					
Year 4	B(.000)	A(.000)			
Cumulative Grade Point Average					
2-2.5					170.98, 9, 0.36, **p<0.001**
2.6-3	B(.000)<br>C(.000)	A(.000)	A(.000)		
3.1-3.5	C(.004)		A(.004)<br>D(.017)	C(.017)	
≥ 3.6	B(.000)<br>C(.001)<br>D(.000)	A(.000)	A(.001)	A(.000)	
Reasons of Studying	C(.000)	C(.000)	A(.000)<br>B(.000)<br>D(.003)	C(.003)	25.30, 3, 0.14, **p<0.001**
Family Responsibility	B(.003)	A(.003)			18.98, 3, 0.12, **p<0.001**
Financial Assistance	B(.022)	A(.022)			13.93, 3, 0.10, **p=0.003**
Scholarship from Government	C(.027)		A(.027)		10.57, 3, 0.09, **p=0.01**
Scholarship from University/Hospital	B(.000)<br>D(.001)	A(.000)		A(.001)	50.21, 3, 0.20, **p<0.001**

*Pairwise Z-Tests with Bonferroni adjustment

Supplementary Table S2. Summary of One-way ANOVA and Post hoc Tukey test. (Only significant pairs were shown)

Post Hoc Tests<br><br>Study Scales	University Nursing (N=1070)<br><br>(A)	University Medical Laboratory Science (N=133)<br>(B)	University Radiography (N=65)<br><br>(C)	University Optometry (N=52)<br><br>(D)	One-way ANOVA
PANAS					
PAS					F(3, 1316)=0.90, p=0.44
NAS	D(.002)	D(.001)		A(.002)<br>B(.001)	F(3, 1316)=5.13, **p=0.002**
GSE	C(.005)		A(.005)<br>D(.007)	C(.007)	F(3, 1316)=5.89, **p=0.001**
CAMS-R	D(.017)	D(.006)		A(0.017)<br>B(.006)	F(3, 1316)=4.10, **p=0.007**
CD-RISC					F(3, 1316)=2.15, p=0.09
BCS					
Self-distraction					F(3, 1316)=1.49, p=0.22
Active coping					F(3, 1316)=1.51, p=0.21
Denial	B(.020)<br>C(.009)	A(.020)	A(.009)		F(3, 1316)=5.76, **p<0.001**
Substance use	C(<.001)	C(<.001)	A(<.001)<br>B(<.001)<br>D(<.001)	C(<.001)	F(3, 1316)=10.90, **p<0.001**
Use emotional support					F(3, 1316)=2.48, p=0.06
Use instrumental support	B(.028)<br>D(.024)	A(.028)		A(.024)	F(3, 1316)=5.30, **p=0.001**
Behavioral disengagement	C(.035)		A(.035)		F(3, 1316)=2.87, **p=0.04**
Venting					F(3, 1316)=1.24, p=0.29
Positive reframing					F(3, 1316)=1.01, p=0.39
Planning					F(3, 1316)=0.94, p=0.42
Humor					F(3, 1316)=1.62, p=0.18
Acceptance	D(.014)			A(.014)	F(3, 1316)=3.31, **p=0.02**
Religion	D(.005)			A(.005)	F(3, 1316)=5.06, **p=0.002**
Self-blame					F(3, 1316)=1.78, p=0.15

Note: Positive and Negative Affect Scale (PANAS); General Self-Efficacy Scale (GSE); Cognitive and Affective Mindfulness Scale, Revised (CAMS-R); Connor-Davidson Resilience Scale (CD-RISC); Brief Cope Scale (BCS).

*Tukey's HSD (honestly significant difference) test

Supplementary Table S3. Univariate linear regressions to determine the correlation between independent variables and resilience in all students and four student groups (N=1320).

Variables	**Total (N=1320)**			**Nursing (N=1070)**			**Medical Laboratory Science (N=133)**			**Radiography (N=65)**			**Optometry (N=52)**		
	B	95% CI	p	B	95% CI	p	B	95% CI	p	B	95% CI	p	B	95% CI	p
Age	-0.38	-0.73, -0.03	**0.03***	-0.34	-0.72, 0.04	0.08	0.19	-0.95, 1.32	0.74	-1.66	-4.18, 0.85	0.19	-1.18	-3.87, 1.51	0.38
Gender<br>Male<br>Female	-0.51	-1.17, 0.14	0.12	-0.78	-1.53, -0.04	**0.04***	1.24	-<br>0.68, 3.15	0.21	-0.25	-3.93, 3.43	0.89	-1.03	-4.92, 2.87	0.60
Marital Status<br>Single<br>Non-single	0.23	-2.30, 2.76	0.86	0.48	-2.16, 3.11	0.72	NA[2]	NA	NA	-1.89	-11.23, 7.46	0.69	NA[2]	NA	NA
Year of Study	-0.20	-0.48, 0.07	0.15	-0.27	-0.56, 0.02	0.07	0.96	0.08, 1.85	**0.03***	NA[1]					
Cumulative Grade Point Average	0.15	-0.25, 0.55	0.47	0.11	-0.36, 0.57	0.65	0.15	-1.03, 1.32	0.80	0.55	-2.00, 3.10	0.67	-0.83	-2.80, 1.15	0.41
Living<br>With family<br>Alone	-0.17	-1.31, 0.98	0.77	-0.25	-1.49, 0.99	0.69	-1.55	-5.87, 2.78	0.48	3.47	-3.18, 10.11	0.30	-1.64	-7.12, 3.84	0.55
Religious Beliefs<br>Yes<br>No	0.56	-0.11, 1.22	0.10	0.49	-0.22, 1.20	0.17	0.95	-1.15, 3.04	0.37	-2.05	-6.17, 2.07	0.32	4.38	-0.02, 8.77	0.05
Reasons of Studying<br>Multiple reasons<br>One of the reasons	0.60	-0.07, 1.27	0.08	0.52	-0.22, 1.25	0.17	1.62	-0.63, 3.87	0.16	-0.38	-3.60, 2.84	0.82	2.84	-1.82, 7.51	0.23
Family Responsibility<br>Dependent<br>No	0.39	-0.37, 1.16	0.31	-0.13	-1.01, 0.75	0.77	1.28	-0.72, 3.27	0.21	3.21	-0.48, 6.90	0.09	1.36	-2.74, 5.46	0.51

Financial Assistance<br>More than one assistance<br>No	-0.53	-1.12, 0.06	0.08	-0.67	-1.31, -0.03	**0.04***	-0.18	-2.08, 1.72	0.85	0.48	-2.93, 3.88	0.78	0.99	-2.97, 4.96	0.50
Scholarship (Government)<br>Yes<br>No	0.07	-1.51, 1.66	0.93	0.53	-1.37, 2.43	0.59	1.90	-2.42, 6.22	0.39	-3.13	-8.68, 2.42	0.26	-1.73	-8.71, 5.25	0.62
Scholarship<br>(University/Hospital)<br>Yes<br>No	0.54	-0.52, 1.59	0.32	0.82	-0.53, 2.16	0.23	-1.19	-3.33, 0.95	0.27	0.21	-5.02, 5.45	0.94	2.61	-2.07, 7.28	0.27
Paid Job<br>Yes<br>No	0.49	-0.12, 1.09	0.12	0.42	-0.24, 1.08	0.21	0.36	-1.52, 2.24	0.71	-2.14	-5.37, 1.09	0.19	5.60	2.11, 9.09	**0.002****
GSE	0.90	0.84, 0.96	**<0.001****	0.89	0.83, 0.96	**<0.001****	0.83	0.64, 1.01	**<0.001****	0.90	0.56, 1.24	**<0.001****	1.32	1.02, 1.62	**<0.001****
CAMS-R	0.92	0.86, 0.99	**<0.001****	0.92	0.85, 1.00	**<0.001****	0.81	0.61, 1.02	**<0.001****	1.19	0.79, 1.60	**<0.001****	1.00	0.58, 1.42	**<0.001****
PANAS															
PAS	0.32	0.28, 0.37	**<0.001****	0.34	0.30, 0.39	**<0.001****	0.19	0.06, 0.32	**0.004****	0.15	-0.70, 0.38	0.17	0.66	0.39, 0.92	**<0.001****
NAS	-0.25	-0.29, -0.22	**<0.001****	-0.24	-0.28, -0.20	**<0.001****	-0.33	-0.42, -0.23	**<0.001****	-0.21	-0.38, -0.04	**0.02***	-0.30	-0.55, -0.06	**0.02***

BCS															
Self-distraction	0.89	0.61, 1.16	<0.001**	0.81	0.49, 1.12	<0.001**	1.32	0.53, 2.10	0.001**	-0.06	-1.61, 1.50	0.94	1.56	0.28, 2.85	0.02*
Active coping	2.04	1.72, 2.36	<0.001**	1.99	1.64, 2.34	<0.001**	2.15	1.22, 3.09	<0.001**	2.44	0.75, 4.13	0.005**	2.07	0.50, 3.64	0.01*
Denial	2.41	2.16, 2.65	<0.001**	2.44	2.17, 2.71	<0.001**	1.80	1.08, 2.51	<0.001**	2.68	1.52, 3.85	<0.001**	3.11	1.68, 4.53	<0.001**
Substance use	-0.15	-0.36, 0.06	0.17	-0.20	-0.43, 0.04	0.10	0.19	-0.49, 0.87	0.58	-0.39	-1.33, 0.55	0.41	0.50	-1.27, 2.26	0.57
Use emotional support	0.78	0.54, 1.02	<0.001**	0.82	0.55, 1.09	<0.001**	0.34	-0.41, 1.10	0.37	0.29	-0.12, 1.69	0.69	1.37	0.23, 2.50	0.02*
Use instrumental support	0.77	0.53, 1.00	<0.001**	0.78	0.52, 1.05	<0.001**	0.61	-0.03, 1.25	0.06	0.49	-0.82, 1.80	0.46	1.34	0.17, 2.51	0.03*
Behavioral disengagement	-0.70	-0.93, -0.48	<0.001**	-0.78	-1.03, -0.53	<0.001**	-0.18	-0.85, 0.48	0.59	-1.16	-2.35, 0.04	0.06	-0.52	-1.85, 0.81	0.43
Venting	0.09	-0.18, 0.36	0.50	0.11	-0.18, 0.41	0.46	-0.06	-0.78, 0.67	0.88	-0.08	-1.58, 1.41	0.91	0.29	-1.43, 2.02	0.74
Positive reframing	2.09	1.83, 2.35	<0.001**	2.21	1.92, 2.50	<0.001**	1.94	1.25, 2.63	<0.001**	1.47	-0.15, 3.09	0.07	1.06	-0.64, 2.76	0.22
Planning	1.32	1.02, 1.61	<0.001**	1.45	1.13, 1.78	<0.001**	0.36	-0.48, 1.20	0.40	1.82	0.15, 3.49	0.03*	1.01	-0.63, 2.65	0.22
Humor	1.50	1.29, 1.70	<0.001**	1.48	1.25, 1.71	<0.001**	1.31	0.73, 1.90	<0.001**	1.75	0.43, 3.07	0.01*	2.01	0.76, 3.25	0.002**
Acceptance	1.92	1.63, 2.21	<0.001**	2.17	1.84, 2.49	<0.001**	0.70	-0.12, 1.52	0.10	1.66	0.11, 3.21	0.04*	1.85	0.53, 3.17	0.01*
Religion	0.44	0.27, 0.62	<0.001**	0.40	0.21, 0.60	<0.001**	0.69	0.17, 1.21	0.01*	0.69	-0.42, 1.80	0.22	0.40	-0.86, 1.65	0.53
Self-blame	-0.67	-0.90, -0.44	<0.001**	-0.59	-0.85, -0.34	<0.001**	-0.85	-1.47, -0.24	0.01*	-0.69	-2.07, 0.69	0.32	-1.34	-2.57, -0.12	0.03*

Note: NA[1] the students are in the same year of study, statistics cannot be computed; NA[2] no students

Positive and Negative Affect Scale (PANAS); General Self-Efficacy Scale (GSE); Cognitive and Affective Mindfulness Scale, Revised (CAMS-R); Connor-Davidson Resilience Scale (CD-RISC); Brief Cope Scale (BCS); *p<0.05; **p<0.01.
